# Epigenetic alterations in head and neck cancer: a brief update

**DOI:** 10.1007/s00428-026-04528-9

**Published:** 2026-04-29

**Authors:** Henrik Hellquist, Pedro Castelo-Branco, Göran Stenman, Alfons Nadal, Abbas Agaimy, Nina Zidar, Reydson Alcides de Lima-Souza, Fernanda Viviane Mariano, Andrés Coca-Pelaz, Alfio Ferlito

**Affiliations:** 1 Faculty of Medicine and Biomedical Sciences (FMCB), University of Algarve, Gambelas Campus, Bld. 2, 8005-139 Faro, Portugal; 2 Algarve Biomedical Center Research Institute (ABC-RI), University of Algarve, Faro, Portugal; 3 Department of Cellular Pathology, Northern Lincolnshire and Goole NHS Foundation Trust, Lincoln, UK; 4 Champalimaud Research Program, Champalimaud Centre for the Unknown, 1400-038 Lisbon, Portugal; 5 Department of Laboratory Medicine/Pathology, Sahlgrenska Center for Cancer Research, Sahlgrenska University Hospital, University of Gothenburg, Gothenburg, Sweden; 6 Pathology Department, Department of Clinical Fundamentals, Universitat de Barcelona, IDIBAPS, Clínic Barcelona, Barcelona, Spain; 7 Comprehensive Cancer Center (CCC) Erlangen-EMN, Institute of Pathology, University Hospital, Friedrich-Alexander University Erlangen-Nürnberg (FAU), Erlangen, Germany; 8 Faculty of Medicine, Institute of Pathology, University of Ljubljana, Korytkova 2, 1000 Ljubljana, Slovenia; 9 Department of Oral Diagnosis, Faculdade de Odontologia de Piracicaba, Universidade Estadual de Campinas (UNICAMP), Piracicaba, São Paulo, Brazil; 10 Department of Pathology, Faculdade de Ciências Médicas, University of Campinas (UNICAMP), Campinas, São Paulo, Brazil; 11 Department of Otolaryngology, Hospital Universitario Central de Asturias, University of Oviedo, ISPA, IUOPA, CIBERONC, Oviedo, Spain; 12 Coordinator of the International Head and Neck Scientific Group, Padua, Italy; 13 University of Udine School of Medicine, Udine, Italy

**Keywords:** Head & neck cancer, Epigenetics, Methylation, Tumour suppressor genes, Clinical implications

## Abstract

DNA methylation of tumour suppressor genes is the most well-studied epigenetic alterations in head and neck cancer. The tumour suppressor genes *CDKN2A, RASSF1,* and *TIMP3* are the most frequently investigated, but the methylation status has been analysed in more than another dozen genes, for example *MGMT*. In oral squamous cell carcinoma (OSCC) methylation of *MGMT, DAPK*, and *CDKN2A* are promising biomarkers of prognostic value. Inhibition of *LSD1*, encoding a histone demethylase, attenuates the development and growth of OSCC. Methylation of *TIMP3* in sinonasal adenocarcinoma (intestinal type) is associated with a significant worse survival, an association not seen in sinonasal squamous cell carcinoma. Olfactory neuroblastoma can be distinguished into four unique subgroups by methylation profiling. Methylation of *RASSF1* is seen in NUT carcinoma, and significantly higher *RASSF1* methylation is found in SMARCB1/INI1-deficient tumours compared to the less aggressive SMARCB1/INI-proficient tumours. Genome-wide methylation profiling in combination with *IDH2* mutation status suggests that tumours with undifferentiable SNUC morphology can be classified into for subgroups. Most salivary gland carcinoma subtypes have specific epigenetic signatures. Four of the most common subtypes, adenoid cystic carcinoma (ADCC), mucoepidermoid carcinoma (MEC), acinic cell carcinoma (ACC), and carcinoma ex pleomorphic adenoma (CXPA) have all methylation of *RASSF1A*, two (MEC and ADCC) also of *TIMP3* and two (MEC and CXPA) of *p16INK*^*4a*^. A methylation landscape of 20 salivary gland tumours (SGTs) is nowadays available.

## Introduction

During the last decades a wealth of studies on genetic aspects of tumour development and progression have led to a tremendous increase in knowledge, particularly regarding mutations, chromosome translocations, and pathways that activate oncogenes and inactivate tumour suppressor and DNA repair genes. Besides genetic changes, epigenetic alterations have increasingly been highlighted as crucial in the initiation and progression of human tumours [1]. Epigenetic alterations refer to stable and heritable changes in gene expression without changes in the DNA sequence [2] and denote the way that genes interact with the environment, in order to generate individual phenotypes [3]. Epigenetic modifications include DNA methylation, covalent histone modifications, chromatin remodelling, and effects of non-coding RNAs and polycomb proteins on gene expression [4–6]*.* DNA methylation is the principal epigenetic factor and is mediated by DNA methyltransferases involving the methylation of cytosine in CpG dinucleotides. CpG islands are regions of DNA found near the start sites of genes (5’ promotor region) and are rich in the dinucleotide sequence "CpG" (cytosine followed by guanine). They have a high guanine-cytosine content compared to the rest of the genome. Hypermethylation of the CpG islands on gene promoters stimulates carcinogenesis, for example via silencing of tumour suppressor genes (TSG) like e.g. *CDKN2A* (p16^INK4a^/p14^ARF^), and *RB1* [7]. Aberrant DNA methylation represents an epigenetic hallmark strongly associated with tumour initiation and progression leading to inactivation or reduced expression of TSGs and activation of oncogenes [8]. Silencing of TSGs, commonly through epigenetic mechanisms like promoter methylation, is a critical early event in many cancers. The methylation status of the hypermethylated oncological region (THOR) of human telomerase reverse transcriptase (*TERT*) has been proven to be a robust diagnostic and prognostic biomarker in a number of human tumours, for example in cancers of the prostate, pancreas and breast, as well as of meningiomas [9–14]. Contrary to gene mutations, epigenetic events are reversible and therefore possible targets for therapeutic intervention.

Head and neck cancer is the seventh most prevalent cancer worldwide accounting for nearly 900,000 new cases annually. The vast majority of cases are squamous cell carcinomas (SCC) including its subtypes and are located in the oral cavity (OSCC), pharynx and hypopharynx, larynx, nasal cavity and its sinuses, and ear [15]. Nearly all cases of SCC in the salivary glands are metastatic from cutaneous SCC [15]. Here we present a brief update on epigenetic alterations in these cancers (Table 1).

**Table 1:** Summary of important gene methylations and their utility

Location & tumour type	Genes	Utility value	
		Prognostic	Diagnostic
Oral SCC	* CDKN2A*	Yes	–
	*DAPK*	Yes	–
	*TIMP3*	Yes	–
	*MGMT*	Yes	–
	*KDM1A*	Yes	–
Nasopharyngeal carcinoma	*RASSF1*	Yes	Yes
Sinonasal malignancies			
Adenocarcinoma (intestinal type, ITAC)	*TIMP3*	Yes	–
NUT	*RASSF1*		
	*BRD4 – NUT*	Yes	–
	(fusion)/EZH2	Yes	Yes
SWI/SNF complex-deficient carcinoma	*SMARCB1*	Yes	–
	*SMARCA4*	Yes	–
	*RASSF1*	Yes	–
SNUC	*IDH2*	Yes	–
Olfactory neuroblastoma	*EDH2, IDH2*	Yes	(Yes)
Salivary gland carcinoma			
MEC, ADCC, ACC, CXPA	*RASSF1*	Yes	–
MEC, ADCC	*TIMP3*	Yes	–
MEC, CXPA	*CDKN2A*	Yes	–
SDC	*ERBB2*	Yes	Yes

### SCC, OSCC and nasopharyngeal carcinoma

A large study of head and neck squamous cell carcinoma (HNSCC) identified 27 aberrantly methylated genes and showed that *FAM135B* methylation (family with sequence similarity 135 member B) was an independent prognostic biomarker for overall survival [16]. The *CDKN2A* gene (cyclin dependant kinase inhibitor 2 A) provides instructions for making the p16(INK4A) and p14(ARF) proteins, both of which function as tumour suppressors. In head and neck cancer *CDKN2A* is one of the most well-studied TSGs with regard to methylation and epigenetic alterations. Mutations in this gene are estimated to be found in 25% of HNSCC. A relatively recent systematic literature review of the epigenetics of head and neck cancers included 25 studies with a total number of 3,790 samples of OSCC and oral potentially malignant disorders. DNA methylation was reviewed in 18 studies and the role of histone modifications was assessed in seven studies. Histone H3 modification, along with *DAPK* (death-associated protein kinase), *TIMP3* (tissue inhibitor of metalloproteinase 3), and *CDKN2A* were the most commonly investigated epigenetic biomarkers. Assessment of the prognostic value of the epigenetic biomarkers was not performed in any of these studies and the clinical applicability of the studies is limited [17, 18]. There are, however, promising ongoing clinical trials such as treatment with low-dose 5-azacytidine.The use of DNMTi 5-Aza for patients with HNSCC sensitizes anti-PD-L1 refractory cancers to reprogramme host immune responses to increase PD-L1 and IFN-γ expression and thereby prolonging the overall survival [19]. In a large review study, the impact of DNA methylation on the risk of oral cancer development was analysed. Promoter methylation of *MGMT* (methylguanine methyltransferase), *DAPK, and CDKN2A* displayed a significant prognostic value and are hence promising biomarkers for oral cancer [20]. Lysine-specific demethylase, LSD1 (encoded by the *KDM1A* gene), is a histone demethylase that is important by promoting cancer initiation as well as cancer progression. Several studies from Manish Bais’s laboratory in Boston have demonstrated the crucial role of LSD1 protein and that inhibition of LSD1 epigenetically attenuates oral cancer development and growth [21–24]. Epigenetic alterations in oropharyngeal SCC reveal distinct differences between HPV-positive and HPV-negative SCC [25, 26]. HPV-negative cancers have a considerably higher degree of genome-wide hypomethylation suggesting that they are far more genomically unstable than HPV-positive cancers which would account for deregulation of cellular processes typical of aggressive neoplasms [27]. Smoking and alcohol have widespread dose dependant epigenetic alterations, primarily via DNA methylation but also histone acetylation, reflecting accelerated biological ageing. Nasopharyngeal carcinoma (NPC) is a rare malignancy in most parts of the world but with a remarkable ethnic and geographic distribution in southern China, Southeast Asia, North Africa, and the Arctic/Alaska. Non-keratinizing squamous cell carcinoma (NK-NPC) is the most common of the three histological subtypes of NPC. The vast majority of NPC are Epstein-Barr Virus (EBV)–associated and EBV acts as an epigenetic driver during NPC pathogenesis [28]. The DNA methylation status of the TSG *RASSF1* can be used as a complement in NPC diagnostics [29]. EBV DNA methylation can also be used in distinguishing NPC from nasal NK/T-cell lymphoma [30].

### Sinonasal malignancies

Newly defined and emerging sinonasal carcinoma entities have different histologies and genomic profiles compared to conventional non-keratinizing and keratinizing SCC, and adenocarcinoma. Epigenetic events in sinonasal SCC and sinonasal adenocarcinoma have been studied well before the new entities were defined. Sinonasal SCC has fewer gene methylation events compared to adenocarcinoma (intestinal-type, ITAC). *RASSF1*, *TIMP3,* and *CDH13* (Cadherin 13) are methylated in both types of carcinomas but have no correlation to clinicopathological features or overall survival in SCCs, contrary to ITAC where *TIMP3* methylation is associated with a significantly worse survival [31].

NUT carcinoma (Nuclear Protein in Testis), SWI/SNF (SWItch/Sucrose Non Fermentable) complex-deficient sinonasal carcinoma (also termed SMARCB1- and SMARCA4-deficient undifferentiated sinonasal carcinoma), and SNUC (Sinonasal Undifferentiated Carcinoma) constitute new carcinoma entities that require the use of molecular identification, mostly detectable by, surrogate antibodies, for classification. SNUC may still be best regarded as a diagnosis of exclusion [32–35]. A fair number of epigenetic studies have been performed on undifferentiated sinonasal carcinomas. Olfactory neuroblastoma (ONB) is a rare sinonasal malignancy and is composed of various cell lineage-specific elements from the olfactory epithelium. Currently there are limited therapeutic options for these cancers. Epigenetic data is relatively limited but methylation profiling of a series of 66 cases of ONB distinguished four unique subgroups. The largest group (64%) had global hypermethylation and one of the smaller groups had a CpG island methylator phenotype (CIMP) and also *IDH2* (Isocitrate Dehydrogenase 2) hotspot mutations [36]. Drugs targeting the mutated forms of *IDH2* are in use for treatment of some cancers associated with these mutations. A relatively recent literature review (articles before 2020) of the genomics and epigenetics of ONB was published in 2021 [37]. The Polycomb group enzyme *EZH2* (Enhancer of Zeste 2) is a critical component of a multiprotein complex with an important role in catalysing the trimethylation of lysine 27 on histone H3 which leads to silencing of many genes, including TSGs. The *EZH2* methyltransferase activity in metastatic ONB has been suggested to be higher than that found in primary ONB. ONB tumours with low *EZH2* repression signature and high *EZH2* activity may be susceptible to treatment with *EZH2* inhibitors, possibly increasing the susceptibility to immunotherapy [38, 39].

SWI/SNF complex-deficient carcinomas have loss of one SWI/SNF complex gene, *SMARCB1* and *SMARCA4* being the most relevant in head and neck cancer [40]. A statistically significant higher methylation of the TSG *RASSF1* (Ras association domain family member 1) has been demonstrated in SMARCB1/INI1-deficient compared to SMARCB1/INI1-positive tumours [41]. *RASSF1* methylation has also been demonstrated in a study of NUT carcinoma where all other 23 genes investigated were unmethylated [42]. Studies of 25 TSGs in sinonasal carcinomas revealed significantly higher DNA methylation in *GATA5, THSB1* (encodes the thrombospondin-1 protein), and *PAX5* genes, and methylation in five or more genes was associated with impaired overall survival [43]. At least two excellent studies on potential DNA methylation-based classification of sinonasal undifferentiated carcinomas have been proposed. *IDH2* mutations in sinonasal carcinomas induce a hypermethylator phenotype (SNUC and large cell neuroendocrine carcinoma) distinct from small cell neuroendocrine carcinoma and SMARCB1-deficient sinonasal carcinomas. Tumours with SNUC morphology may not be as undifferentiated as previously considered and may be divided into at least three classes, possibly four. Two of these (having *IDH2* or *SMARCA4/ARID1A* mutations) with more favourable clinical course than the highly aggressive SMARCB1-deficient carcinomas [44, 45].

### Salivary gland cancer

This is a very heterogeneous group of neoplasms, albeit being rare, and where the malignant epithelial tumours currently are histologically classified into 21 different subtypes. They often have overlapping histologies, different genomic profiles and biological behaviour. Due to their rarity and diversity personalized treatment can be difficult to evaluate. The important role of epigenetic alterations in cancer initiation and development has been recognised for several decades and some initial studies were performed already 20 years ago. These studies included for example promoter methylation of *CDKN2A, RASSF1A* and *DAPK* in adenoid cystic carcinoma (ADCC). A high frequency of *RASSF1A* methylation was noticed in high-grade tumours and in tumours with metastasis, suggesting that methylation of this gene may play an important role in the progression of ADCC [46–48]. Mucoepidermoid carcinoma (MEC) showed hypomethylation of *CLIC3* (coding chloride intracellular channel protein 3) but no correlation with clinical or pathological characteristics [49]. Another study investigated the prevalence of promotor methylation of *CDKN2A, TP53* and *TERT* genes in MEC. The highest value was for *CDKN2A* and interestingly *TERT* was more often methylated in lower clinical stages [49`]. Two comprehensive reviews on epigenetic alterations in salivary gland cancer were published 3–5 years ago summarizing the methylated genes in the most common salivary carcinomas. For example, *RASSF1A* was methylated in MEC, ADCC and acinic cell carcinoma (ACC), and in carcinoma-ex-pleomorphic adenoma (CXPA), *TIMP3* in MEC and ADCC, and CDKN2A in MEC and CXPA [50, 51]. In a review of 47 studies of different salivary gland tumours (SGT) meta-analysis revealed that both *MGMT* (methylguanine methyltransferase) and *RASSF1A* were methylated and related to higher stages of the tumours but not associated with the grade of the tumours [52]. A comprehensive study of 363 cases encompassing 20 SGT entities provided a DNA methylation landscape of SGTs. Most entities had specific epigenetic signatures, cribriform adenocarcinoma being epigenetically distinct from polymorphous adenocarcinoma and myoepithelioma and pleomorphic adenoma (PA) formed their own unique class [53]. There is currently insufficient evidence to prove a link between SGTs, HPV infection and DNA methylation of TSGs [54]. Studies of *TERT* promoter (THOR) methylation in parotid PA, recurrent PA, CXPA and in adjacent histologically normal parotid tissue indicate that THOR methylation in recurrent PA is closer to carcinoma than to normal tissue. A subset of PA-adjacent tissues showed epigenetic alterations, possibly suggestive of increased risk of recurrence [55]. Another recent study used DNA methylation profiling for differentiating between PA and CXPA. Three distinct clusters, benign, intermediate and malignant, were identified. Pathogenic mutations of *TP53, HRAS, PTEN* and/or *TERT* were only present in CXPA [56].

## Summary

Epigenetics is an evolving science within the biomedical field. The main difference between the molecular pathology of gene changes and epigenetic alterations is that the latter are reversible offering new therapeutic possibilities. Methylation profiling can be used as a complement in for example distinguishing adamantinoma-like Ewing sarcoma from conventional Ewing sarcoma [57] and nasopharyngeal carcinoma from nasal NK/T cell lymphoma [30]. Within head and neck cancer, a number of recurrent epigenetic alterations with clinical implications both with regard to diagnosis, prognosis, and therapy, have recently been demonstrated and several clinical trials are now ongoing.

## Data Availability

Data supporting the findings of this study are available within the article.
